# Convalescent Spontaneous Coronary Artery Dissection With Elevated Pericoronary Fat Attenuation Index

**DOI:** 10.1016/j.jaccas.2026.108128

**Published:** 2026-06-03

**Authors:** Alec L. Sinatro, Thomas J. Nero, Sharonne N. Hayes

**Affiliations:** a Department of Internal Medicine, Northwell Health, Lenox Hill Hospital, New York, New York, USA; b Department of Cardiology, Maine Health, USA; c Department of Cardiovascular Medicine, Mayo Clinic, USA

**Keywords:** coronary circulation, dissection, fibromuscular dysplasia, hybrid imaging, imaging, intravascular ultrasound, myocardial infarction, myocardial ischemia

## Abstract

**Background:**

Myocardial infarction with nonobstructive coronary arteries (MINOCA) is defined by the presence of the universal acute myocardial infarction criteria in the absence of obstructive disease on angiography. Multimodal imaging can be invaluable in determining the underlying etiology of MINOCA. Spontaneous coronary artery dissection (SCAD) is among the most common etiologies.

**Case Summary:**

This report describes a case of unrecognized/asymptomatic myocardial infarction due to SCAD in an active 62-year-old woman whose care was facilitated by comprehensive evaluation of MINOCA.

**Discussion:**

Pericoronary fat attenuation indexing (FAI) has yet to be extensively studied in the setting of MINOCA. Initial studies suggest elevated pericoronary FAI in MINOCA and SCAD, providing introductory evidence that vascular insult increases coronary artery inflammation appreciably via pericoronary FAI.

**Take-Home Message:**

This case report further substantiates that claim and demonstrates that coronary computed tomography angiography with pericoronary FAI may prove useful in working up MINOCA.

## Graphical Abstract

**Figure:**
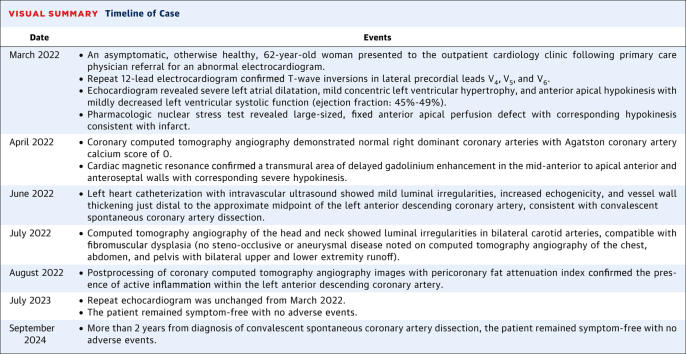

## History of Presentation

A 62-year-old woman was referred to the outpatient cardiology clinic following an abnormal electrocardiogram (ECG). She had seen her primary care physician for a routine checkup several days prior, and the ECG demonstrated new T-wave inversions in the lateral leads. She reported no chest pain/pressure, nausea, vomiting, diaphoresis, or dyspnea. She had recently retired and started high-intensity endurance training, including daily long-distance cycling and rowing. She reported no limitations in her activities and felt well overall.

**Take-Home Messages**

• In the setting of MINOCA, coronary CTA with pericoronary FAI may determine whether infarcted myocardium occurred secondary to vascular insult within a particular segment of coronary artery, as in the diagnosis of SCAD.
• This information may then guide subsequent studies to ensure that a diagnosable underlying condition is not missed.

On examination, the patient appeared healthy and younger than her stated age. Vital signs were within normal limits: heart rate 57 beats/min; blood pressure 130/80 mm Hg bilaterally. Body mass index was 24.30. Physical examination was unremarkable, aside from well-healed bilateral breast surgical scars.

## Past Medical History

Past medical history was significant for ductal carcinoma in situ treated with double mastectomy and reconstructive breast surgery in 2011 and hyperlipidemia managed with high-intensity statin therapy. History was negative for hypertension, diabetes, smoking, and use of supplements or illicit substances.

Family history was significant for sudden death of her sister at age 47 believed by the patient to have been due to “aortic dissection.” On further questioning and subsequent acquisition of her sister's autopsy report highlighting the presence of hemorrhagic dissection in the proximal left anterior descending coronary artery (LAD) resulting in extensive myocardial necrosis, a distinct, aorta-unrelated differential diagnosis was proposed: atherosclerotic coronary artery disease with intraplaque hematoma or spontaneous coronary artery dissection (SCAD). After her sister's death, the patient was evaluated and had normal ECG, aorta, and left ventricular function assessed via echocardiography.

## Differential Diagnosis

Differential diagnosis included atherosclerotic coronary artery disease with or without intraplaque hematoma, SCAD, Takotsubo cardiomyopathy, coronary vasospasm, infiltrative myocardial disease, and focal myocarditis.

## Investigations

Lipid panel demonstrated total cholesterol 230 mg/dL, low-density lipoprotein cholesterol 104 mg/dL, high-density cholesterol 116 mg/dL, triglycerides 35 mg/dL with normal lipid subfractionation, lipoprotein (a) <10 nmol/L, and high-sensitivity C-reactive protein <0.3 mg/L.

A 12-lead ECG demonstrated T-wave inversions in the lateral precordial leads V4, V5, and V6 (Figure 1). Subsequent echocardiogram revealed severe left atrial dilatation, mild concentric left ventricular hypertrophy, anterior apical hypokinesis, and mildly decreased left ventricular systolic function with an ejection fraction of 45% to 49%. No aortic dilatation or valvular pathology was noted.

**Figure 1:**
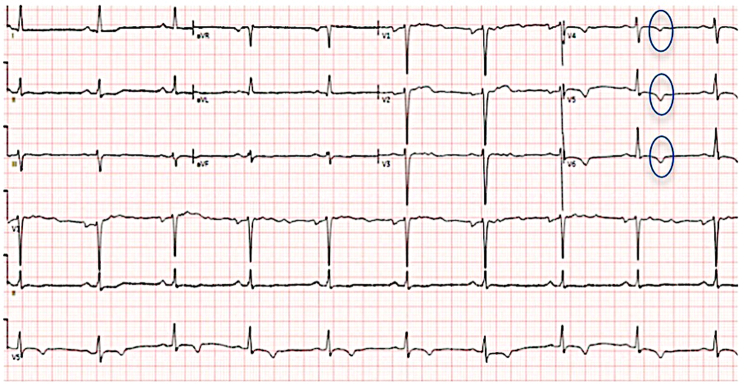
Electrocardiogram A 12-lead electrocardiogram demonstrates T-wave inversions in lateral precordial leads V_4_, V_5_, and V_6_ (encircled).

Pharmacologic nuclear stress test with regadenoson perfusion and sestamibi imaging was subsequently performed, revealing a large-sized, severe intensity, fixed anterior apical perfusion defect with corresponding hypokinesis consistent with infarct. Gated coronary computed tomography angiography (CTA) was subsequently performed, demonstrating normal right dominant coronary arteries with Agatston coronary artery calcium score of 0. Considering the discordance in findings between coronary CTA and nuclear imaging, cardiac magnetic resonance was obtained, which demonstrated a moderately sized, transmural area of delayed gadolinium enhancement in the mid-anterior to apical anterior and anteroseptal walls with corresponding severe hypokinesis (Figure 2). Cardiac catheterization with intravascular ultrasound (IVUS) was scheduled for precise visualization and potential intervention in the setting of evident infarction and impaired contractility.

**Figure 2:**
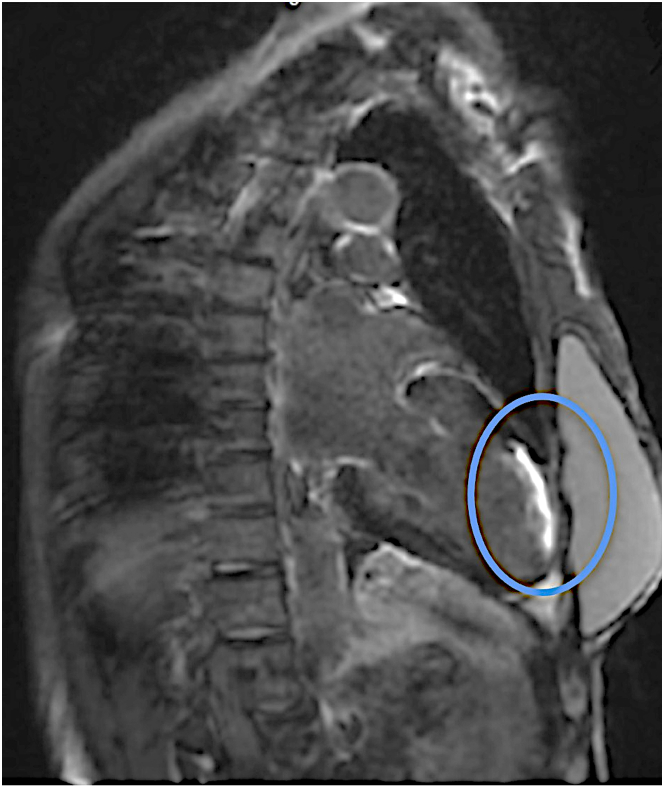
Cardiac Magnetic Resonance Cardiac magnetic resonance with phase-sensitive myocardial delayed enhancement demonstrates a moderately sized transmural area of delayed gadolinium enhancement in the mid-anterior to apical anterior and anteroseptal walls, consistent with infarct (encircled area).

Left heart catheterization demonstrated mild luminal irregularities in the distribution of the LAD with an instantaneous wave-free ratio of 0.93 at the apex. Coronary vasoreactivity was tested according to the 2019 European Society of Cardiology guidelines^1^: simultaneous ECG and instantaneous wave-free ratio testing were performed with each injection, without evidence of spasm. IVUS failed to demonstrate diffuse coronary atherosclerosis or intimal dissection flap; however, increased echogenicity and significant thickening of the vessel wall were noted just distal to the approximate midpoint of the LAD (Video 1). Intramural characteristics represent 2 of the most common findings suggesting fibrosis and chronic remodeling in the setting of convalescent SCAD.^1^ It is worth noting that although optical coherence tomography offers higher resolution and thus more precise characterization of intramural pathology relative to IVUS, it was not pursued in this case due to concern surrounding substantially increased risk of iatrogenic dissection extension from high-pressure contrast injection in a potentially SCAD-affected artery. Instead, IVUS was employed as a potentially safer yet still diagnostically informative alternative.

Considering the described constellation of findings, convalescent SCAD emerged as the most likely underlying diagnosis. Postprocessing of coronary CTA images with pericoronary fat attenuation index (FAI) subsequently confirmed the presence of active inflammation within the LAD with a weighted FAI score of 16.8 (−56.6 HU) (98th percentile) (Figure 3).

**Figure 3:**
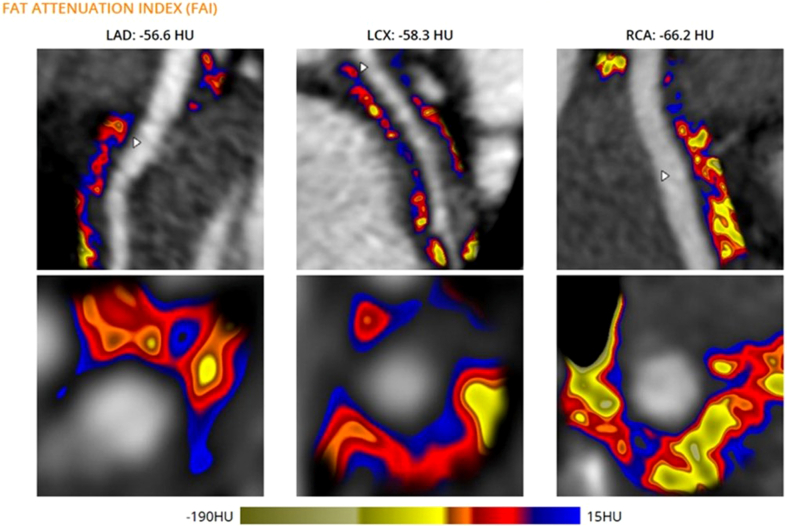
Coronary Computed Tomography Angiography Coronary computed tomography angiography with pericoronary fat attenuation index (FAI) demonstrates significant inflammation (multicolored areas) most pronounced throughout the left anterior descending coronary artery (LAD) (shown in the coronal plane), as well as throughout the left circumflex coronary artery (LCX). RAD = right coronary artery.

CTA of the head and neck demonstrated luminal irregularities in bilateral carotid arteries, compatible with fibromuscular dysplasia. No steno-occlusive or aneurysmal disease was noted on CTA of the chest, abdomen, and pelvis with bilateral upper and lower extremity runoff.

## Management, Outcome, and Follow-Up

In the 2 years since coronary angiography, the patient has continued to be highly active and symptom-free. Her most recent echocardiogram was performed 15 months after her prior evaluation and was unchanged. The patient's blood pressure remains within the desirable range without medical intervention. She remains on rosuvastatin 20 mg, which was initiated 2 years before diagnosis of SCAD. She does not take any antiplatelet or anticoagulant therapy.

## Discussion

Our case report highlights a unique case of myocardial infarction with nonobstructive coronary arteries (MINOCA). SCAD is a relatively rare form of myocardial infarction that is now being recognized with increasing frequency. Review of the MINOCA literature reveals a diagnosis of SCAD in up to 20% of patients.^2^ The current etiology of SCAD appears to be acute medial hemorrhage followed by luminal narrowing or dissection. Though long-term outcomes are generally favorable, registries have demonstrated recurrence rates of 11% to 20%.^3^ The precise underlying cause of hemorrhage has yet to be fully elucidated. Coronary CTA has gained traction as a potentially useful tool to aid in the diagnosis of MINOCA; nevertheless, published reports on coronary CTA findings in both acute and convalescent SCAD have only recently been added to the armamentarium.^3^ As the precise and definitive role of FAI in SCAD remains under evaluation, preliminary studies indicate increased pericoronary inflammation that is not limited to the dissected artery.^4^ Importantly, and as was the case in the index patient, increased pericoronary inflammation can be appreciated via pericoronary FAI in the setting of otherwise normal coronary imaging and proinflammatory biomarker analysis.

Our evaluation highlights interesting questions regarding the evaluation of MINOCA and SCAD. Though coronary CTA undoubtedly plays a role, it can often be misleading when used as the sole source of diagnostic criteria, as many of the characteristic findings (eg, acute dissection flap) have proven difficult to discern on coronary CTA due to its relatively low spatial resolution. Diagnosing convalescent SCAD presents its own set of unique challenges inherent to the rapid healing that often takes place following dissection. Partially healed arteries can be misdiagnosed as normal.^5^ Accordingly, full evaluation for the etiology of MINOCA is essential to accurately diagnose and appropriately tailor both acute and long-term management. In this case, the diagnosis of SCAD led to appropriate comprehensive evaluation for fibromuscular dysplasia and other extracoronary arteriopathies.^6^

A significant limitation of this case is the absence of invasive coronary angiography during the acute phase. Invasive coronary angiography is considered the gold standard for the acute diagnosis of SCAD, and its absence inevitably introduces diagnostic uncertainty. However, this limitation reflects a real-world clinical scenario: in the present case, invasive coronary angiography was not performed acutely because the patient remained asymptomatic and unaware of any acute coronary pathology until incidental electrocardiographic changes were identified during routine screening. By the time invasive angiography was performed, characteristic findings of dissection flaps and/or intramural hematomas often encountered in cases of acute SCAD were absent. Instead, signs of significant intramural healing were noted in the affected segment, complicating the definitive differentiation of convalescent SCAD from other potential underlying etiologies, such as intraplaque hematoma. Nevertheless, the characteristic findings of increased echogenicity and persistent vessel wall thickening seen on IVUS are well-described features of resolving intramural hematoma and fibrotic healing post-SCAD—particularly in the setting of an otherwise atheroma-free coronary tree, as was the case in this patient.

**Visual Summary:** Timeline of Case

Date	Events
March 2022	• An asymptomatic, otherwise healthy, 62-year-old woman presented to the outpatient cardiology clinic following primary care physician referral for an abnormal electrocardiogram. • Repeat 12-lead electrocardiogram confirmed T-wave inversions in lateral precordial leads V_4_, V_5_, and V_6_. • Echocardiogram revealed severe left atrial dilatation, mild concentric left ventricular hypertrophy, and anterior apical hypokinesis with mildly decreased left ventricular systolic function (ejection fraction: 45%-49%). • Pharmacologic nuclear stress test revealed large-sized, fixed anterior apical perfusion defect with corresponding hypokinesis consistent with infarct.
April 2022	• Coronary computed tomography angiography demonstrated normal right dominant coronary arteries with Agatston coronary artery calcium score of 0. • Cardiac magnetic resonance confirmed a transmural area of delayed gadolinium enhancement in the mid-anterior to apical anterior and anteroseptal walls with corresponding severe hypokinesis.
June 2022	• Left heart catheterization with intravascular ultrasound showed mild luminal irregularities, increased echogenicity, and vessel wall thickening just distal to the approximate midpoint of the left anterior descending coronary artery, consistent with convalescent spontaneous coronary artery dissection.
July 2022	• Computed tomography angiography of the head and neck showed luminal irregularities in bilateral carotid arteries, compatible with fibromuscular dysplasia (no steno-occlusive or aneurysmal disease noted on computed tomography angiography of the chest, abdomen, and pelvis with bilateral upper and lower extremity runoff).
August 2022	• Postprocessing of coronary computed tomography angiography images with pericoronary fat attenuation index confirmed the presence of active inflammation within the left anterior descending coronary artery.
July 2023	• Repeat echocardiogram was unchanged from March 2022. • The patient remained symptom-free with no adverse events.
September 2024	• More than 2 years from diagnosis of convalescent spontaneous coronary artery dissection, the patient remained symptom-free with no adverse events.

## Conclusions

The precise role of pericoronary FAI in the comprehensive evaluation after MINOCA is not firmly established. It has shown promise in highlighting active coronary inflammation as an etiology and prognostic biomarker in the setting of coronary atherosclerosis.^7^^,^^8^ Subsequent studies will thus serve to determine if pericoronary FAI shows comparable utility in the evaluation of MINOCA and diagnosis of SCAD in the setting of otherwise nondiagnostic coronary imaging.^9^^,^^10^

## Funding Support and Author Disclosures

The authors have reported that they have no relationships relevant to the contents of this paper to disclose.

## Appendix

Video 1 Cardiac Catheterization Cardiac catheterization with high-definition intravascular ultrasound demonstrates increased echogenicity and significant thickening of the vessel wall most pronounced just distal to the approximate midpoint of the left anterior descending coronary artery.
